# Development and Testing of Advanced Cork Composite Sandwiches for Energy-Absorbing Structures

**DOI:** 10.3390/ma12050697

**Published:** 2019-02-27

**Authors:** Paweł Kaczyński, Mariusz Ptak, Fábio A. O. Fernandes, Leszek Chybowski, Johannes Wilhelm, Ricardo J. Alves de Sousa

**Affiliations:** 1 Faculty of Mechanical Engineering, Wroclaw University of Science and Technology, Łukasiewicza 7/9, 50-371 Wrocław, Poland; pawel.kaczynski@pwr.edu.pl (P.K.); johannes.wilhelm@pwr.edu.pl (J.W.); 2 TEMA: Centre for Mechanical Technology and Automation, Department of Mechanical Engineering, University of Aveiro, Campus de Santiago, 3810-193 Aveiro, Portugal; fabiofernandes@ua.pt; 3 Faculty of Marine Engineering, Maritime University of Szczecin, Waly Chrobrego 1-2, 70-500 Szczecin, Poland

**Keywords:** cork, composites, graphene, mechanical tests, crashworthiness, energy-absorbing materials, natural materials

## Abstract

Cork is a sustainable material with remarkable properties. In addition to its main application as wine stoppers, it has also been employed as a sound and thermal insulator in facades, building roofs, aeronautical applications, and, more recently, in impact energy absorption systems. In its natural form, cork is mainly used in wine stopper manufacturing, but for other applications, cork compounds are usually employed, which makes it possible to manufacture complex geometries with nearly isotropic behavior. In this work, an attempt was made to merge the desirable properties of two different cork materials (agglomerated and expanded black) into cork composite sandwich structures. These structures were tested according to impact conditions typically experienced by energy-absorbing liners used in personal safety devices. Additionally, the performance dependency on the working temperature was analyzed. The sole black, expanded cork (EC159) and agglomerated cork (AC199A and AC216) were tested in 500 J impacts. It was found that black cork was characterized by superior thermal stability, while expanded cork allowed absorbing high energies. In the second stage, the composites consisting of both tested materials were tested in 100 J impact scenarios. The combination of two materials of different properties enabled reduction of the peak force exerted on a helmet user’s head during the impact by about 10% compared to agglomerated specimens. Additionally, it was proved that there was no influence of the glue used to join different cork types.

## 1. Introduction

Many human activities carry a high risk of hard impacts and require the responsible use of special safety equipment. For example, head and neck injuries are the main causes of disability and deaths among motorcycle and bicycle users [1], and protection from these types of accidents requires a portable and wearable solution. One of the most common safety devices is a helmet, which is the most crucial part of protective gear for motorcyclists, racing drivers, downhill skiers, etc. [2,3]. A helmet protects the head on many levels, including protection from bruises to lowering the severity of an impact by reducing the peak acceleration of the brain.

The materials in helmets are developed to absorb and dissipate energy by converting kinetic energy to some other form of energy by undergoing plastic or viscoelastic deformation, friction, etc. A material can be considered a decent energy absorber if it manages to cushion a user’s head by keeping its deceleration below a critical threshold value, specified by either regulations or virtual head models [4,5,6]. The most popular selections of engineered materials used as the inner layer of helmets are foams and honeycomb structures [7,8]. Cellular materials like foams are well-known for their excellent energy-absorbing capabilities and are commonly used in industry to manufacture passive safety devices [9,10]. When compressed, their cellular structure exhibits elastic and plastic bending and/or crushing of the cell walls [11,12,13]. During this phase, energy can be absorbed at a near-constant stress level (plateau stress). Aside from the absolute amount of energy absorbed, isotropic behavior of a material is preferred to ensure a uniform behavior under varying loads [14,15,16,17]. If multi-impact scenarios are possible, the ability of material to recover after the initial impact should also be considered [18,19].

Cork may be an alternative material for both foams and honeycomb structures. While cork is mainly used as a wine stopper material, its properties have been noticed, and it is currently used in various applications from flooring underlayment to thermal protection systems on heavy-lift rockets [20,21,22,23]. Its exceptional properties include a high damping factor and excellent acoustic and thermal insulation [24,25], as well as a very low permeability to liquids and gases, chemical stability, and durability [26,27]. In addition, it has an attractive appearance and a naturally superior touch and feel, which makes it desirable for premium products—jewelry, bags, wallets, and automotive design elements [28,29,30]. The fact that it is renewable and recyclable fits well within the current environmental consciousness of potential customers. In contrast, other cellular materials used in similar applications are typically synthetic foams, whose production can cause environmental problems since most plastics take many years to degrade [31]. 

Currently, cork is produced in two forms—natural and agglomerated. To produce natural cork, the bark of a cork oak tree is stripped every eight to nine years [32]. First, cork is boiled to eliminate insects, bacteria, and fungi, as well as to make it softer and more flexible. Then, it rests one to six months to stabilize the moisture content, and then the final shape of a product can be cut out of the bark strips. Natural cork, after being harvested and stabilized, shows anisotropic behavior, resulting in mechanical responses that are dependent on the loading direction, which is an issue for its use in passive safety devices. 

Agglomerated cork (AC) is created by milling natural material into granules of a desired size and subsequently cleaning and drying the granules, followed by mixing them with polyurethane (PU) thermosetting resins. Then, the mixture is placed into a mold, pressurized, and heated to temperatures between 110 °C and 150 °C, which causes agglutination of granules. Agglomerated cork consists of randomly-oriented granules connected by a binder, which display a quasi-isotropic response [33]. It is worth noting that an agglomerated material can be produced in any size and shape. An additional advantage of agglomerated cork over the natural form is the ability to tailor its properties during the manufacturing phase by changing the size of the granules, the type of the binder, or the mixing ratio. This fact combined with the more homogeneous properties of the agglomerated form make it a better candidate for use in passive safety devices [7,18,19,34,35,36,37,38].

The excellent properties of cork—together with its sustainability—attracted the attention of some researchers who developed environmentally-friendly composite structures [39]. Composite structures based entirely on natural materials (more specifically, agglomerated cork, natural fibers, and bio-based binders) were developed by Fernandes et al. [40]. The natural fibers used were obtained by compounding flax fibers with bio-resin and agglomerated cork as the core materials. In addition to the advantage of eco-friendliness, the results showed that these structures can compete against synthetic solutions. Similar studies have been performed, but the samples in these studies were only tested at quasi-static rates and with an epoxy matrix [41]. Urbaniak et al. [42] also studied agglomerated cork as a core material in sandwiches with glass-epoxy laminates. Additionally, researchers have also developed cork composite filaments for fused deposition modeling [43] and cork-polymer composites for injection molding [39,44].

A special kind of agglomerated cork is expanded cork (EC), which is also known as the “black agglomerate”. It was developed to provide a fully-natural material made from the bark of oak tree branches that were previously unused [20]. This part of the trees has the greatest concentration of extractives, which allows them to be used as natural adhesives. High amounts of natural resin results in a much darker color of the final product. The grinding stage is identical for white agglomerates. In the next step, the bark is milled into granules, and then the granules are enclosed in autoclaves where a pressure around 40 kPa is applied. Simultaneously, the temperature is elevated to 300–370 °C for 20–30 min. The natural resin (suberin) is exuded outside the granules, which start to expand, and agglomeration proceeds without any additives. Cork cell walls contain predominantly suberin in association with lignin, a lesser content of polysaccharides, and an even lower level of extractives [45]. Suberin is a lipophilic biopolymer greatly responsible for cork’s properties such as its impermeability to water and gases. Suberin is practically infusible, and it is insoluble in water, alcohol, ether, chloroform, concentrated sulphuric acid, and hydrochloric acid, among others. Expanded cork is fully recyclable, reusable, and biodegradable, and it has also been found that medium-density EC is also resistant to long-term exposure to external conditions [46,47].

**Figure 1:**
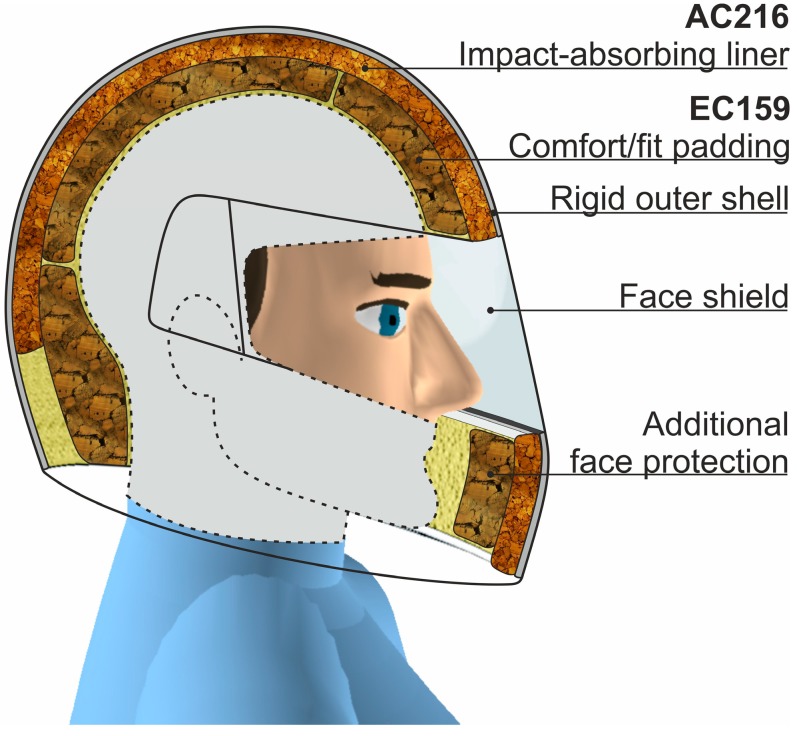
The cross-section of a motor helmet where cork sandwiches have been applied.

There has been extensive research performed to determine how well each type of cork can absorb impact energy [19,34,35,48]. The results have suggested that cork is well-suited to play a role as an impact energy-absorbing liner in a helmet. A possible use for both types of cork agglomerates could lead to better helmet designs with superior energy absorption during an accident. The use of the EC cork may increase the comfort and safety of the user by transferring an impact force through a larger area, while the outer AC material may help to absorb high-energy impacts. Figure 1 depicts the cross-section of a motor helmet where the advanced cork composite sandwich is applied. 

The behavior of the cork was checked under both static and dynamic conditions. Fernandes et al. [19] compared expanded polystyrene (EPS) and agglomerated cork to determine their performance in impact scenarios and concluded that cork materials could be used as absorption liners in passive safety devices. Regarding the widely varying working environments of passive safety devices—namely different initial energy levels and temperature ranges from −30 °C to 100 °C—agglomerated cork material with a density of 216 kg/m^3^ showed potential to replace the widely-used EPS [35,48]. The influences of strain rate and adhesives were also studied.

The dynamic tests performed on AC were conducted on a drop test machine during which the mechanical response of the materials was investigated at a broad range of impact energies. Sanchez et al. [49] tested a relatively low maximum energy of 46.7 J with a 4.753 m/s impact velocity. Jardin et al. [34] tested a moderate impact energy of 115.2 J with a 4.8 m/s impact velocity, which is more relevant when considering the use of cork as an energy-absorbing liner in a motorcycle helmet designed in accordance with the ECE R22.05 standard. Ptak et al. [48] tested both AC and EC at a very broad range of impact energies (120–850 J) and velocities (5.2–13.7 m/s) to simulate the most demanding applications of helmets where cork padding was used for extreme sports [48]. During the tests, it was discovered that AC is more stable than its expanded form. Even at a moderate rate of impact energy spectrum (120 J), samples of expanded cork were damaged, which limited their use as reliable, high-level impact, and multi-impact energy absorbers. On the other hand, this property of EC, which tended to crush under a lower load than EC, may also be seen as an advantage in terms of head injury mitigation. 

Kaczynski et al. [35] tested the effects of temperature on the energy absorption of three cork agglomerates, AC199A, AC216, and EC159. The goal was to check whether higher temperatures would have negative effects on the binder used in the micro agglomerates. The difference in the absorbed energy in most cases was relatively small (below 10%), with one exception—a lower energy absorption of AC216 subjected to an 850 J impact. The authors explained this behavior by the less uniform glue distribution of AC216 compared to AC199A (which coped well with the 850 J impact under the same conditions) caused by the bigger, less uniform grain size of AC216. A less uniform distribution of a binder means a less uniform heat transfer during high-energy impact, which in turn causes the binder to experience exceedingly high local heating and results in a reduction of its adhesive performance.

## 2. Materials and Methods

Currently, advanced passive safety devices are designed with energy absorption liners that consist of a few materials with different properties—this trend is reported in motorcycle helmets, for example. This is done to achieve progressive energy-absorption behavior that greatly influences the injury level of the brain. Hence, in addition to testing the specimen out of one micro-agglomeration, the authors decided to examine a combination of different cork materials and selected five different cork types based on the knowledge acquired from earlier studies [35,48]. The monomaterial specimen’s height was 60 mm, and the cross-section was set as 50 × 50 mm. To keep the specimen’s height constant, the sandwich specimens consisted of two monomaterial elements with the same cross-section but with only a 30 mm height. The higher one (closer to the impactor) was always EC159, and the lower one (on the anvil) was AC216 or AC199A. Thus, the following nomenclature was established: X_Y, where X is the material closer (top) to impactor and Y is on the anvil (bottom). Soudal 22A cork glue with an acrylic dispersion base was used to connect the EC samples with a 30 mm height with other AC samples of the same size. The thickness of the applied glue layer measured approximately 0.5 mm. During the glue curation, a constant 50 N force was applied. The thermal resistance of the glue after drying was between −20 °C to +80 °C, and its specific weight was 1.5 g/cm^3^. An overview of the tested materials is presented in Table 1.

The temperature of tested specimens was set by putting them into a climatic test chamber, which allowed the authors to precisely control temperature. The climatic test chamber was equipped both with heaters (allowing temperatures up to 100 °C) and with nitrogen cooling connectors (for temperatures of −30 °C, −15 °C, and 0 °C). The chamber was equipped with a flow system to guarantee a uniform temperature distribution during testing. The temperature observation always took place by using K-type thermocouples and measuring the core temperature of each specimen.

To characterize the energy-absorption behavior of the agglomerated cork specimens, dynamic impact tests were performed using a Dynatup 9250 HV (Instron, Norwood, MA, USA) spring-loaded drop-weight tower. The device was equipped with a circular-flat tup with a 50 mm diameter and a total mass of 11.2 kg. To achieve desired energy levels of 100 J and 500 J, different impact velocities were set. Using an optical gate placed few millimeters above the specimen, impact velocities were measured as v = 4.1 m/s and 9.2 m/s.

The tested specimens were placed in the center of the lower anvil, and the sampling rate of a load cell installed between the falling mass and the tup was set to 204.8 kHz. The sensors of the drop hammer allowed only for direct registration of force signals, and the deflection signal may only have been obtained through a double integration of the acquired signal. Due to the limited accuracy of this approach, each specimen drop test was additionally recorded by a Phantom V12 (Vision Research, Wayne, NJ, USA) high-speed camera with a frame rate of 10,000 fps. The position of high-contrast markers was placed beforehand on the lower anvil, and the tup was also tracked by TEMA motion analysis software (Image Systems Motion Analysis, Linköping, Sweden). This allowed the authors to determine the specimen’s deflection signal at a sample rate of 10 kHz.

To make the sampling rate of both signals equal, LabVIEW software (LabVIEW 2014, National Instruments, Austin, TX, USA) was used to upscale the deflection signal by performing a “spline interpolation” algorithm. Furthermore, the deflection signal was correlated in time to be consistent with the force signal.

## 3. Results and Discussion

The compression process where the specimens were subjected to a high-energy impact of 500 J is shown in Table 2. The video data were gathered by recording the procedure with a high-speed Phantom V12 camera. The specimens shown in Table 2 reached their maximum compressive displacement approximately 14 ms after the initial contact of the specimen and the tup and undergo large strain deformation. Cork material is known to have a Poisson’s ratio of approximately 0–0.15, and its cell walls show mainly elastic buckling effects during the compression process up to densification. The time sequence is shown here for two different agglomerated cork types and one expanded cork type, as well as their combinations, namely AC199A, AC216, EC159, as well as EC159_AC199A and EC159_AC216 at 24 °C.

As was previously reported by the authors in [35], the crashworthiness of agglomerated cork is greatly influenced by temperature. Here, the samples tested at a temperature of 100 °C were less stiff and showed a lower energy absorption. The cracking of the AC216 material was observed, and the side walls of the specimen delaminated. At a temperature of −30 °C, the stiffness of the specimen was greatly increased, and no cracking occurred.

On the other hand, EC159 cork material and composites that included EC159 cork material could not exhibit the same compression behavior, and the EC had no additional binder to influence crashworthiness as a function of the testing temperature. Furthermore, expanded cork material generally loses its integrity under impact easier than agglomerated cork, which results from the use of a suberin that connects the grains as a natural but weak binder.

**Figure 2:**
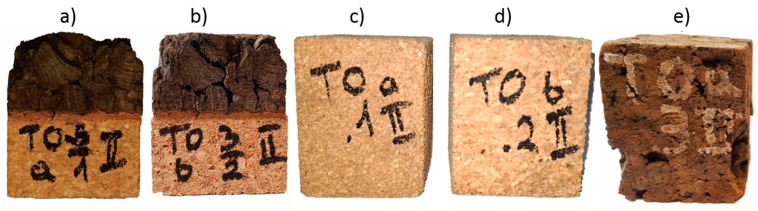
The cork samples after the 100 J impact at 24 °C (**a**) EC159_AC199A, (**b**) EC159_AC216, (**c**) AC199A, (**d**) AC216, (**e**) EC159.

Previous results reported by Kaczynski et al. in [35] were analyzed by examining the thermal stability of the black agglomerate. The minimum deflection during all 500 J impacts was equal to 40 mm, thus for comparison purposes, a deflection of 38 mm was set as a threshold. Moreover, during standalone testing, EC159 material was unable to absorb such a high energy and did not stay intact (Figure 2). Additionally, during all tests, the test stand was equipped with bumpers to prevent tup-to-anvil contact for deflections greater than 45 mm. 

Analysis of the force registered during the 500 J impact on the black agglomerate shows that bumpers were touched, and the force level increased rapidly from a value of about 2–3 kN to about 60 kN after 45 mm of deflection. The force level after 45 mm of deflection increased due to the contact between the falling mass and bumpers, thus it could not be attributed to the cork properties and was always discarded.

### 3.1. Finding 1: EC159 is a Thermally-Stable Material for Impact Compared to Agglomerated Cork

The most important conclusion drawn from the experiment is that expanded cork is a thermally-stable material whose crashworthiness is not greatly affected compared to the most commonly used agglomerated cork types. In the case of black cork, the difference between the maximum energy absorbed (122 J for *T* = 0 °C) and the minimum energy absorbed (56 J for 100 °C) was equal to 66 J. In the case of the AC216 cork type, the difference was about 4.93 times larger and equaled 326 J (406 J for −30 °C and 80 J for 100 °C). The lack of temperature sensitivity was also confirmed in [35] on the basis of a CAMEA (cork agglomerates model for energy absorption) model defined as:

(1) $$E\left(d\right)=A\cdot d^{B}\cdot C^{T}$$

where *d*—specimen deflection (mm), *T*—specimen temperature (°C), *A*, *B*, *C*—variables (-).

**Figure 3:**
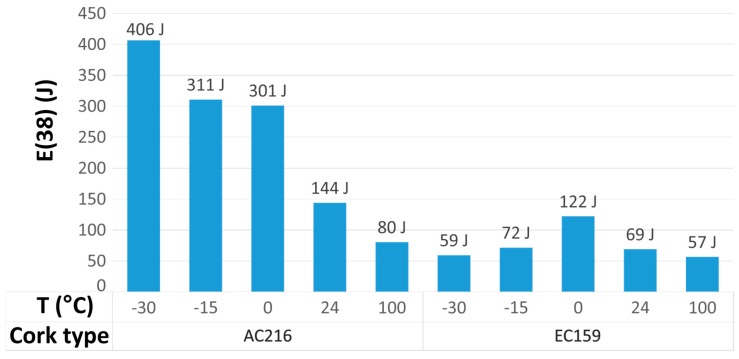
The influence of temperature on the absorbed energy at 38 mm deflection after 500 J impact for AC216 and EC159 based on [35].

The C coefficient presented in Table 3 of the CAMEA model of EC159 cork was almost equal to one, which indicated a lack of temperature sensitivity. However, EC159 cork absorbed much less energy compared to AC216, but in terms of its use as inner comfort padding in helmets, this should not be regarded as a disadvantage. The influence of the temperature on energy absorbed is presented in Figure 3.

The maximum energy-absorbing capabilities of the EC159 material were observed at 0 °C (Figure 3). The crashworthiness was reduced when the temperature was either increased or decreased because of the nature of the EC159 binder, which is a natural resin. An increase in the cell wall thickness at negative temperatures [50] caused an increase in the compressive strength, since the buckling of cell walls became more difficult. On the other hand, sub-zero temperatures resulted in changes of the lignin and suberin amount [51]. The phenolic cross-linking between cell wall polymers, deposition of lipids on the cell wall, and extension on the cell wall during cold acclimation may have also increased rigidity [52]. In contrast, an increase in the temperature reduced the resin viscosity and adhesive capabilities.

### 3.2. Finding 2: The Crashworthiness of the Sandwich Cork is Better than the Monomaterial

**Figure 4:**
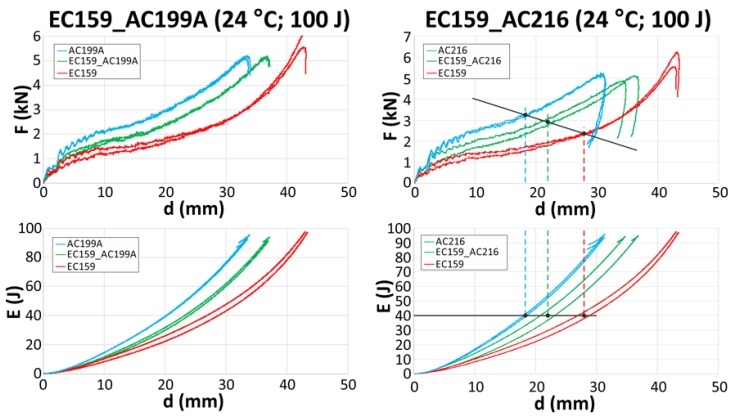
Force-displacement (upper row) and energy-displacement (lower row) curves of tested cork sandwiches for EC159_AC199A (left column) and EC159_AC216 (right column).

Once the cork samples during high energy impacts (500 J) and high strain rates were tested, the authors studied the EC159 combined with much stiffer materials (AC216 and AC199A) against lower energy impacts of 100 J. Such an energy level was selected to better reproduce a real impact condition. The use of EC159 as a top material slightly decreased the energy-absorbing capabilities in both cases (EC159_AC216 and EC159_AC199A). The deflection needed to absorb a 100 J impact increased by about 4 mm. The behavior of the sandwich samples is presented in Figure 4. The force-displacement and energy-displacement curves of the sandwich samples lay in the middle of the curves for each standalone material. One of the most crucial criteria for determining the severity of head injuries is the acceleration level, which is directly proportional to the force and is independent of the type of head protection used (e.g., a bike or motorcycle helmet). Therefore, a way to reduce the maximum force level is a primary goal. In the case where the energy level equaled 40 J, the deflection of the material accordingly equaled 18.5 mm for AC216, 21.5 mm for EC159_AC216, and 28 mm for EC159. The maximum forces registered for these deflections were equal to 3.3 kJ for AC216, 2.9 kJ for EC159_AC216, and 2.4 kJ for EC159.

**Figure 5:**
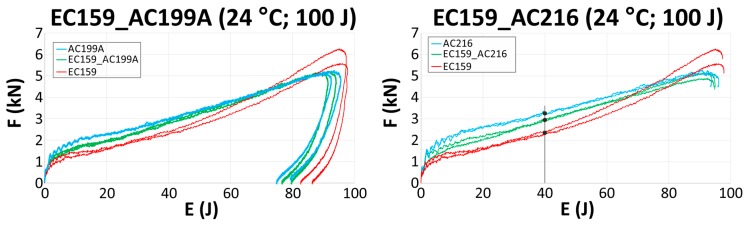
Force-energy curves of tested cork sandwiches.

The force-energy curves of the manufactured sandwich materials are presented in Figure 5. The curves represent the maximum force registered during sandwich crushing as a function of absorbed energy. The effect was visible for both of the tested sandwiches, but it was higher for EC159_AC216, since AC216 is a denser and stiffer material. Despite that, crushing EC159 (Figure 5, red curve) resulted in higher force values than it did for the denser and stiffer AC199A cork agglomerate for energy levels greater than 80 J (Figure 5, blue curve). Adding the relatively soft EC159 to AC216 decreased the maximum crushing force in the whole range of absorbed energies (Figure 5, green curve).

### 3.3. Finding 3: The Glue Interface Does not Influence the Crashworthiness of Specimens

**Figure 6:**
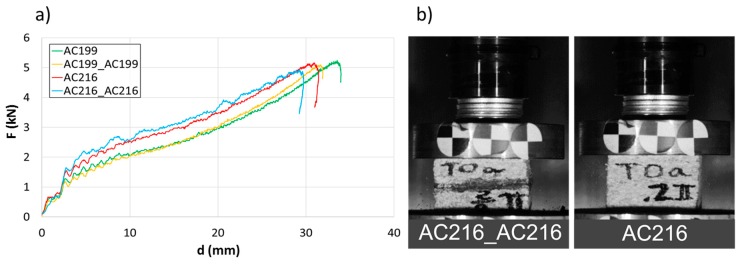
Comparison of mono-material agglomerate and sandwich behavior (**a**) force-deflection curves (**b**) crushing behavior at t = 10 ms.

The character of force-displacement curves depicted in Figure 6a and the observed compression behavior in Figure 6b were also very similar. When analyzing the differences in the deflection of the specimen, a difference of about 1 mm was observed, which may have been due to the accuracy of the specimen cutting process.

There was no influence from the glue used for joining corks. For instance, specimens marked as AC216 after 29 mm of deflection absorbed 105.1 J, while glued specimens, marked as AC216_AC216, absorbed 106.8 J after the same deflection. The use of glue resulted in an almost 1.5% greater energy absorption, which was negligible or within the measurement error. According to the glue manufacturer, this product is thermally stable up to 80 °C, thus the influence of glue on the specimen crashworthiness was only tested at room temperature. 

### 3.4. Finding 4: The Interface Glue Fills Cork Voids at the Micro Level

**Figure 7:**
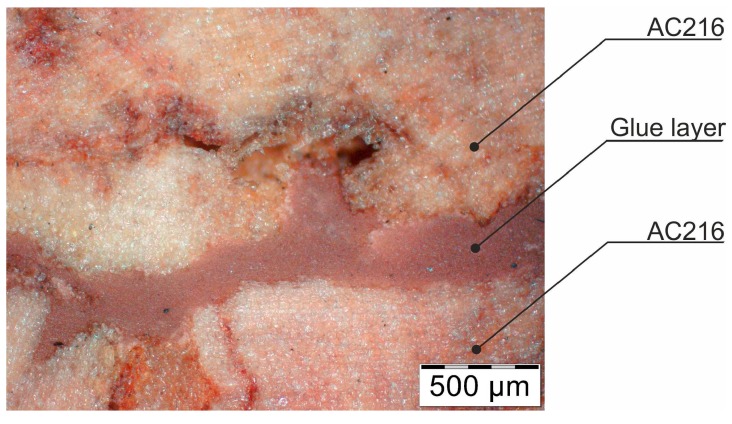
The photograph of the glue layer between AC216 cork outer layers.

The cross-section through the middle part of the specimens is shown in Figure 7. The photographs taken using a stereo microscope show that Soudal 22A glue thoroughly filled the empty spaces between cork granules borders, leaving almost no underfill. 

The glue layer had a thickness of about 0.2–0.5 mm. The glue thickness variation resulted from the rough cutting of corks using a band-saw, which prevented specimens that were cut this way from being smooth. 

## 4. Conclusions

A standard cork agglomerate is composed of bound triturated granules, while expanded cork is made by heating cork granules, which forces them to expand to release their natural components, consequently binding the granules together. A test of the composite structures showed that cork, in conjunction with other materials, could be used to create unique and useful behavior for use in passive safety devices. The decent performance of composites made of expanded cork allows wider design possibilities compared to monomaterial white cork solutions. In addition, it makes it possible to perform further adjustments regarding crashworthiness properties for a given application. Indeed, some applications require gentle decelerations at lower stresses while absorbing impact energy, and others require high energy absorption for more extreme cases. The wise combinations of black and white agglomerates may provide the perfect fit to such demands.

The benefit of the proposed solution lies not only in the alteration of the mechanical properties but also in the additional value of visual attractiveness, since cork is increasingly used in premium products. Further research could be done using different materials such as EPP (expanded polypropylene) foam or expanded cork as the filler of the AC216 sample cut-outs.

A summary of the most important conclusions drawn from the conducted experiments is as follows:

1. EC159 is a thermally-stable material for impact compared to agglomerated cork.
2. The crashworthiness of the sandwich cork is better compared to the monomaterial.
3. The glue interface does not influence the crashworthiness of the specimens.
4. The interface glue fills cork voids at the micro level.

## Figures and Tables

**Table 1:** Material properties and the tested temperatures.

Cork Naming Density [kg/m^3^] Grain Size [mm]	Section	Impact Energy [J]	Temperature [°C]
AC199A 199 0.5–2	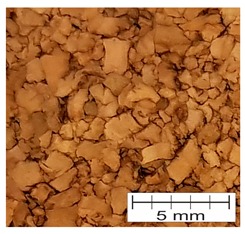	100	24
AC216 216 2–4	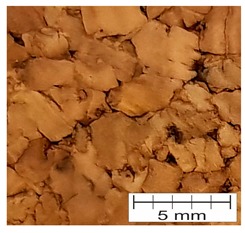	100	24
		500	−30; −15, 0, 24, 100 ^1^
EC159 159 4–10	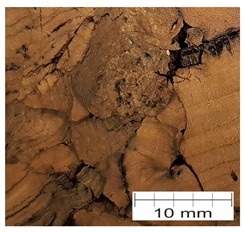	100	24
		500	−30; −15, 0, 24, 100 ^1^
EC159_AC199A n.a. n.a.	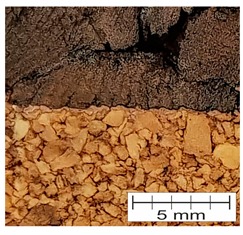	100	24
EC159_AC216 n.a. n.a.	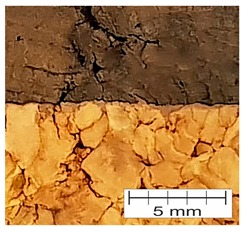	100	24
C216_AC216 n.a. n.a.	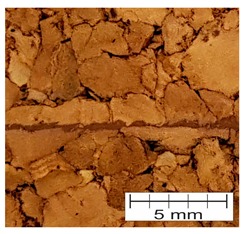	100	24

^1^ The behaviour of the specimen under 500 J in extreme temperature was published in [35].

**Table 2:** Time sequence of 100 J impact for two different cork sandwiches: EC159 is shown on top and AC199A/AC216 on bottom, and the AC199A, AC216, and EC159 cork samples at 24 °C.

Type	EC159_AC199A	EC159_AC216	AC199A	AC216	EC159
**0.0 ms**	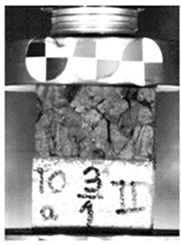	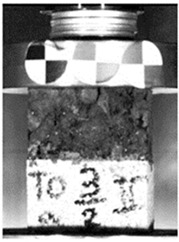	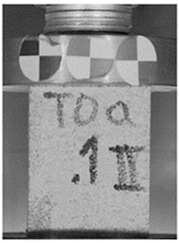	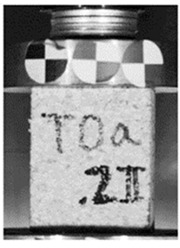	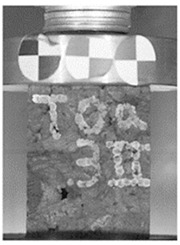
**5.0 ms**	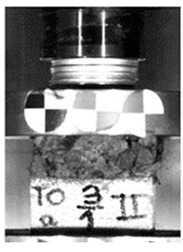	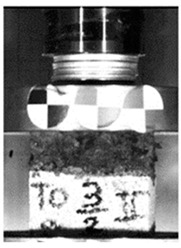	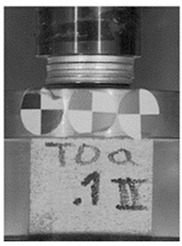	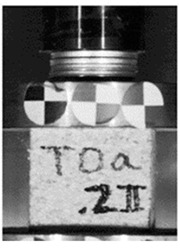	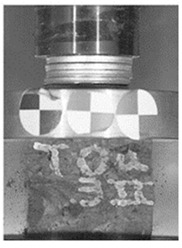
**10.0 ms**	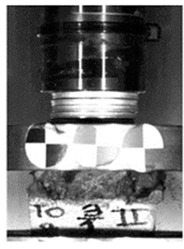	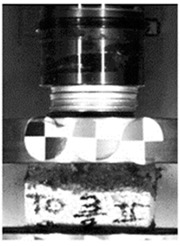	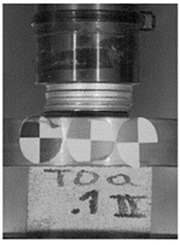	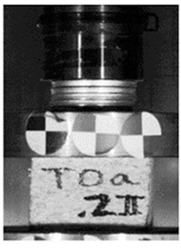	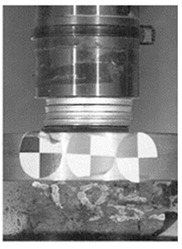
**15.0 ms**	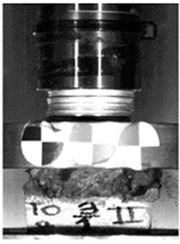	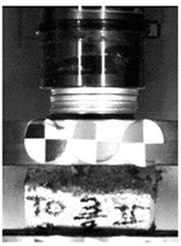	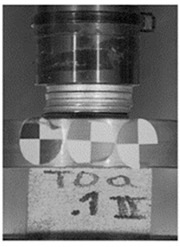	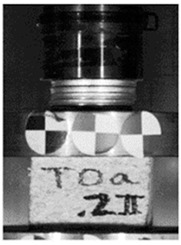	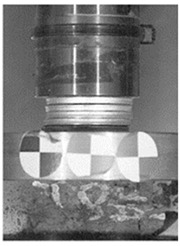

**Table 3:** Variables of the cork agglomerates energy absorption model (CAMEA).

Cork Type	A	B	C
AC216	0.145	2.086	0.988
EC159	0.133	1.786	0.999
